# An Ethanol Extract of *Artemisia iwayomogi* Activates PPARδ Leading to Activation of Fatty Acid Oxidation in Skeletal Muscle

**DOI:** 10.1371/journal.pone.0033815

**Published:** 2012-03-27

**Authors:** Si Young Cho, Hyun Woo Jeong, Jong Hee Sohn, Dae-Bang Seo, Wan Gi Kim, Sang-Jun Lee

**Affiliations:** Health Science Research Institute, Research and Development Center, AmorePacific Corporation, Bora-dong, Giheung-gu, Yongin-si, Korea; Pennington Biomedical Research Center, United States of America

## Abstract

Although *Artemisia iwayomogi* (AI) has been shown to improve the lipid metabolism, its mode of action is poorly understood. In this study, a 95% ethanol extract of AI (95EEAI) was identified as a potent ligand of peroxisome proliferator-activated receptorδ (PPARδ) using ligand binding analysis and cell-based reporter assay. In cultured primary human skeletal muscle cells, treatment of 95EEAI increased expression of two important PPARδ-regulated genes, carnitine palmitoyl-transferase-1 (CPT1) and pyruvate dehydrogenase kinase isozyme 4 (PDK4), and several genes acting in lipid efflux and energy expenditure. Furthermore, 95EEAI stimulated fatty acid oxidation in a PPARδ-dependent manner. High-fat diet-induced obese mice model further indicated that administration of 95EEAI attenuated diet-induced obesity through the activation of fatty acid oxidation in skeletal muscle. These results suggest that a 95% ethanol extract of AI may have a role as a new functional food material for the prevention and/or treatment of hyperlipidermia and obesity.

## Introduction

Skeletal muscle is an important organ in the whole body regulation of energy homeostasis and the main site of fatty acid and glucose oxidation [1], [2]. PPARδ plays a critical role in skeletal muscle metabolism via transcriptional regulation of downstream gene expression [3]. The reported *in vivo* effects of PPARδ activation include improvement of dylipidemia and hyperglycemia, prevention of diet-induced obesity, enhancement of insulin sensitivity and modulation of muscle fiber type switching as demonstrated by systemic ligand administration or by generation of transgenic mice that over-express an active PPARδ [4]–[7]. Most of the observed beneficial effects are believed to be mediated by increasing fatty acid catabolism and mitochondrial function in muscle and adipocytes [8]. Thus, it is proposed that activators of PPARδ may have therapeutic utility in the treatment of metabolic disease [9].

*Artemisia* herbs, a member of the *Compositae*, have long been used in foods and in traditional medicine for treatment of diseases, including diabetes and hepatitis [10]. *Artemisia* herbs have been reported to have anti-diabetic and anti-hyperlipidemic activities in diabetic patients and rats [11], [12]. However, molecular mechanisms whereby *Artemisia* exerts its benefit on lipid and glucose metabolism remain unknown.

In this study, we screened medicinal herbs to search for natural PPARδ ligands. We found that a 95% ethanol extract of *Artemisia iwayomogi* (95EEAI) directly interacted with PPARδ, enhanced the expression of genes involved in lipid catabolism and induced PPARδ-dependent activation of fatty acid oxidation. Furthermore, administration of 95EEAI to mice fed a high-fat diet enhanced fatty acid oxidation in the skeletal muscle and protected against diet-induced obesity.

## Materials and Methods

### Ethics statement

All animal experiments were approved by the AmorePacific Institutional Animal Care and Use Committee and adhere to the OECD guidelines. Permit numbers: AP11-101-FR012. No specific permits were required for the described field studies. No specific permissions were required for these locations/activities. We confirmed that the location was not privately-owned or protected in any way and the field studies did not involve endangered or protected species.

### Preparation of an ethanol extract of *Artemisia Iwayomogi*

Three hundred grams of the aerial parts of *Artemisia iwayomogi* was heated to 80°C with 70% ethanol for 3 h. The extract was then filtered through Whatman No. 1 (Whatman, Piscataway, NJ, USA) and loaded on a D-101 macroporous resin column, followed by elution of the column with water (WEAI), 50% ethanol (50EEAI) and 95% ethanol eluate (95EEAI) were obtained. After evaporation, the solutions were freeze-dried.

### PPARδ coactivator assay

PPARδ coactivator assay was performed using Lanthascreen™ time-resolved fluorescence resonance energy transfer (TR-FRET) PPARδ coactivator assay kit according to the instructions of the manufacturer (Invitrogen, Carlsbad, CA, USA). All assays were validated for their robustness by determining the respective Z′-factors [13]. Measurements were performed on a VICTOR3_V Multilabel Counter (WALLAC 1420; PerkinElmer Life and Analytical Sciences, Rodgau, Germany) with instrument settings as described in the manufacturer's instructions for LanthaScreen™ assays.

### Luciferase Reporter Assay

PPARδ-responsive luciferase reporter assay was performed using Human Peroxisome Proliferator-Activated Receptor Delta Reporter Assay System (Indigo Biosciences, PA, USA). PPARδ reporter cells (provided with assay system) are non-human mammalian cells stably transfected with human PPARδ and PPARδ-responsive luciferase reporter genes. Mock reporter cells which contain only the PPARδ-responsive luciferase vector were also purchased from Indigo Biosciences. PPARδ reporter cells and mock reporter cells were cultured in Cell Recovery Medium 1 (CRM-1) for 4 h. The cells were treated with indicated concentration of AI extracts for 24 h. Luciferase activity was measured using luciferase detection reagent (Indigo Biosciences, PA, USA) and Tecan infinite M200 Pro (Tecan, Grodig, Austria), following the manufacturer's recommended procedures. The protein concentration of the cell lysate was determined using the BCA protein assay kit (Pierce, Rockford, Illinois, USA). Luciferase activity was normalized to the protein concentration of each sample. The fold induction of normalized luciferase activity was calculated relative to DMSO (vehicle)-treated cells, and represents the mean of three independent samples per treatment group.

### Human Primary Skeletal Muscle Cell Culture

Normal human skeletal muscle myoblasts were purchased from LONZA (Walkersville, MD, USA) and cultured in skeletal muscle growth medium (SkBM-2, SkGM-2 SingleQuots, LONZA Walkersville, Inc., MD, USA), 100 units/ml penicillin, and 100 µg/ml streptomycin in a humidified atmosphere of 5% CO_2_. To differentiate human myoblasts into myotubes, cells with a density of 60–70% were grown in Dulbecco's minimum essential medium: Nutrient Mixture F-12 (DMEM/F-12) with 2% horse serum for 5 days. Myotubes were treated with PPARδ agonist (GW501516, Alexis Biochemicals, Lausen, Switzerland), various concentration of AI extracts (WEAI, 50EEAI, 95EEAI) or the vehicle (DMSO) for 24 h.

### Small Interfering RNA Treatment

The PPARδ small interfering RNA (siRNA) pool (a mixture of three siRNAs for PPARδ, cat. no. sc-36305) and control siRNA (cat. no. sc-37007) were purchased from Santa Cruz Biotechnology (Santa Cruz, CA, USA). For the transfection procedure, cells were grown to 70% confluence, and PPARδ and control siRNAs were transfected using the Lipofectamine 2000 reagent (Invitrogen, Carlsbad, CA, USA) according to the manufacturer's instructions. Briefly, Lipofectamine 2000 reagent was incubated with serum-free medium for 10 min. Subsequently, a mixture of the respective siRNAs was added. After incubation for 15 min at room temperature, mixture was diluted with medium and added to each well. The final concentration of PPARδ siRNA in each well was 100 nM. After culturing for 48 h, cells were washed and treated with GW501516 or AI extracts for an additional 24 h.

### Real Time Quantitative RT-PCR

Total RNA was extracted with TRIzol (Gibco-BRL, Invitrogen Corp., Carlsbad, CA, U.S.A.) according to the manufacturer's instructions. The pre-designed primers and probe sets of PPARδ, CPT1β, PDK4, uncoupling protein-3 (UCP3), PPARγ coactivator 1α (PGC1α), acyl-Coenzyme A dehydrogenase, long-chain (LCAD), and glyceraldehyde-3-phosphate dehydrogenase (GAPDH) were obtained from Applied Biosystems (assay ID for human: Hs00987011_m1, Hs00993896_g1, Hs01037712_m1, Hs01106052_m1, Hs01016719_m1, Hs01085277_m1, and Hs03929097_g1, respectively/ assay ID for mouse: Mm01308156_g1 (CPT1β), Mm01166879_m1 (PDK4), Mm00627598_m1 (UCP2), Mm00494077_m1 (UCP3), Mm01208835_m1 (PGC1α), Mm00599660_m1 (LCAD), Mm99999915_g1 (GAPDH)). The reaction mixture was prepared using a Quantitect probe PCR kit (Qiagen, GmbH, Germany) according to the manufacturer's instructions. Reaction and analysis were performed using the Rotor-Gene 3000 system (Corbett Research, Sydney, Australia). All reactions were done in triplicate. The amount of mRNA was calculated by the comparative CT method.

### Western blot analysis

Myotubes were lysed in RIPA buffer (PBS, pH 7.4, containing 1% NP-40, 0.5% sodium deoxycholate, 0.1% SDS, and a protease inhibitor cocktail). Forty micrograms of proteins were resolved on 10% NuPAGE gels run in an MES buffer system (Invitrogen, Carlsbad, CA) and transferred to nitrocellulose membranes according to the manufacturer's protocol. Immunoreactive proteins were revealed by enhanced chemiluminescence with ECL Plus(Amersham, GE healthcare, Buckinghamshire, UK). Antibodies against PPARδ and GAPDH were purchased from Santa Cruz Biotechnology. Blots were analyzed with a LAS-3000 imaging system (Fujifilm, Japan).

### Fatty Acid Oxidation

Myotubes placed in a 12-well plate, or 400 mg of intact mouse quadriceps muscles were washed and incubated in low glucose DMEM (Invitrogen) containing 2% (w/v) fatty acid-free BSA, 0.3 mM L-carnitine, and [^3^H]palmitic acid (3 µCi/well, PerkinElmer Life, Boston, MA, USA). After incubation, medium was precipitated with an equal volume of 10% trichloroacetic acid (Sigma) by centrifugation at 12,000 rpm for 10 min.. The supernatant was extracted twice with chloroform/methanol (2∶1) and then counted for ^3^H_2_O production.

### Glucose Uptake

Differentiated primary human muscle myotubes were incubated in serum-free, low-glucose DMEM containing 0.1% BSA for 16 h at 37°C. Cells were treated with GW501516, AI extracts and vehicle for 24 h at 37°C and then stimulated with or without 100 nM insulin for 1 h at 37°C. Glucose uptake was initiated by the addition of 2-deoxy-D-[^14^C]glucose (PerkinElmer Life) at a final concentration of 3 µmol/l for 10 min in HEPES buffer-saline (140 mM NaCl, 5 mM KCl, 2.5 mM MgCl_2_, 1 mM CaCl_2_, 20 mM HEPES, pH 7.4). The reaction was terminated by separating cells from the HEPES buffer saline and 2-deoxy-D-[^14^C]glucose. After three washes in ice-cold PBS, the cells were extracted with 0.1% SDS and subjected to scintillation counting for ^14^C radioactivity. The protein concentration was determined with a BCA assay kit (Pierce, Rockford, IL, USA), and the radioactivities were normalized by determining each total protein concentration.

### Animal Experiments

C57BL/6J mice (male, 5 weeks of age) were purchased from Samtako (Osan, Korea). The mice were housed individually in a temperature- and humidity-controlled (26.5°C and 35%) facility with a 12-h light/dark cycle. All animals were allowed free access to water and diets. After acclimatization for 1 week, mice were fed a normal chow diet (D12450B, Research Diets, New Brunswick, NJ, USA) or a high-fat diet (60% kcal fat, D12492, Research Diets). Food consumption and body weight were recorded every week. A 95EEAI was given at 200 mg/kg of body weight by oral zoned needle once daily for 8 weeks. At the end of the experiment, blood and quadriceps muscle tissue samples were collected from the mice. Plasma triacylglycerol, total cholesterol and total ketone bodies were measured by an automated TBA-120FR biochemical analyzer (Toshiba) by using commercial assay kits (Dai-ichi Pure Chemicals, Tokyo, Japan). Plasma-free fatty acids and blood glucose levels were determined by a NEFA C-test (catalog no. 279-75401, Wako Pure Chemical, Osaka, Japan) and GLU neo SINO-Test (SINO-Test, Tokyo, Japan), respectively. Serum level of β-hydroxybutyrate was measured with high sensitivity and specificity according to the manufacturer's directions (Autokit 3 β-hydroxybutyrate, Wako Diagnostics, Richmond, VA) by calculating the rate of Thio-NADH (β-thionicotinamide adenine dinucleotide) production spectrophotometrically at 405 nm upon oxidation of 3- β-hydroxybutyrate.

### Statistical Analysis

Experiments were performed at least three times. The data were reported as a mean ± S.D. Results were analyzed by Student's *t*-test or ANOVA using the program SPSS 11.0 (SPSS, Chicago, IL, USA). Statistical significance was set at *p*≤0.05.

## Results

### A 95EEAI is PPARδ ligand

**Figure 1:**
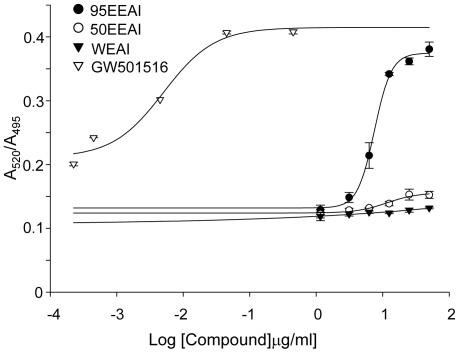
Ligand-induced binding of a coactivator derived peptide to PPARδ *in vitro*. Interaction of fluorescein-labeled coactivator peptide C33 and recombinant GST-PPARδ bound by a terbium-labeled anti-GST antibody was determined by TR-FRET. GW501516, WEAI, 50EEAI and 95EEAI were used at the concentrations indicated. Results are expressed as the ratio of fluorescence intensity at 520 nm (fluorescein emission excited by terbium emission) and 495 nm (terbium emission). All data points represent averages of triplicates (±S.D.).

**Figure 2:**
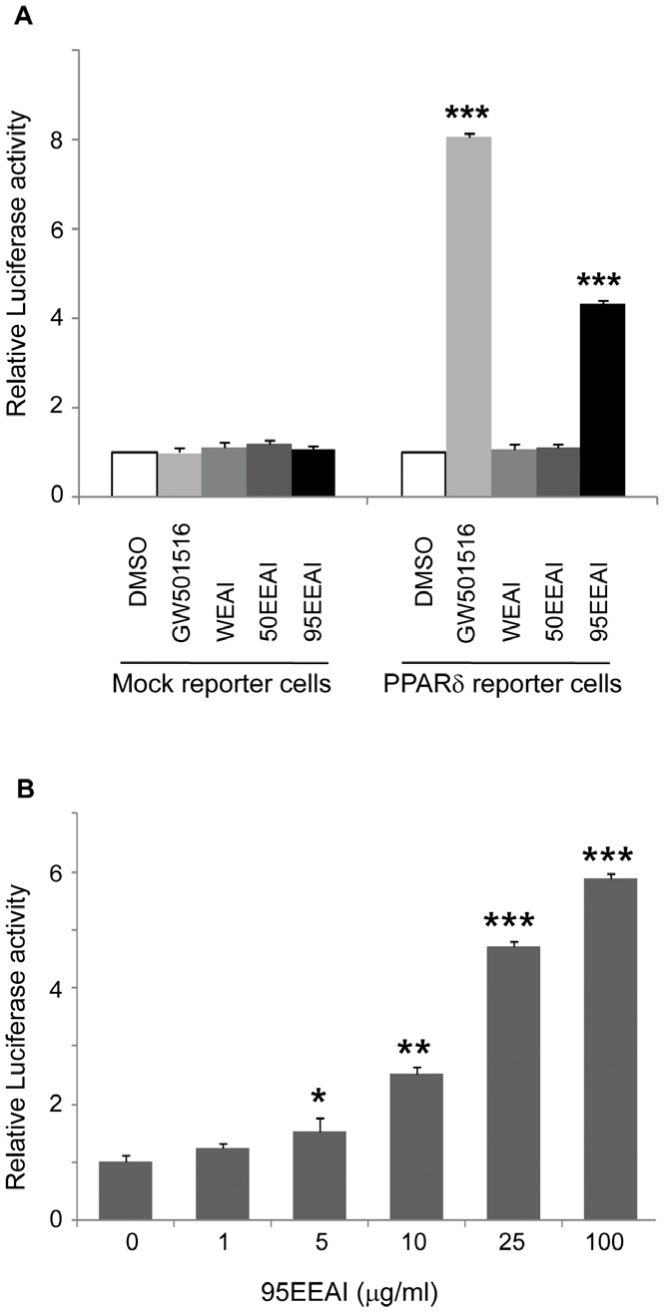
Effects of 95EEAI on the transcriptional activity of PPARδ reporter cells. (A) Mock reporter cells (luciferase vector only) and the PPARδ reporter cells (PPARδ expression vector+PPARδ-responsive luciferase reporter vector) were cultured, treated with DMSO, GW501516 (1 µM), WEAI (100 µg/ml), 50EEAI (100 µg/ml) and 95EEAI (100 µg/ml) for 24 h before harvesting. (B) The effects of various ranges of 95EEAI concentrations were analyzed. Values represent averages of six independent experiments (± S.D.). ***, p<0.005; **, p<0.01 compared to vehicle control.

We found that a 95EEAI interacted with the PPAR δ ligand binding domain (LBD) (EC_50_ = 7 ug/ml, Z′-Factor = 0.65, Figure 1) in repeated assay. We then determined the ability of a 95EEAI to activate PPARδ using cell-based PPARδ-responsive luciferase reporter assays. The synthetic PPARδ agonist GW501516 (1 µM) caused a strong luciferase activity (Figure 2A). Similarly, a 95EEAI induced the luciferase activity in a dose-dependent manner (Figure 2B), while WEAI and 50EEAI had no effect (Figure 2A). Altogether, these results show that a 95EEAI is capable of activating PPARδ via interaction with LBD of PPARδ.

### A 95EEAI induces the expressions of PPARδ target genes in a PPARδ-dependent manner

**Figure 3:**
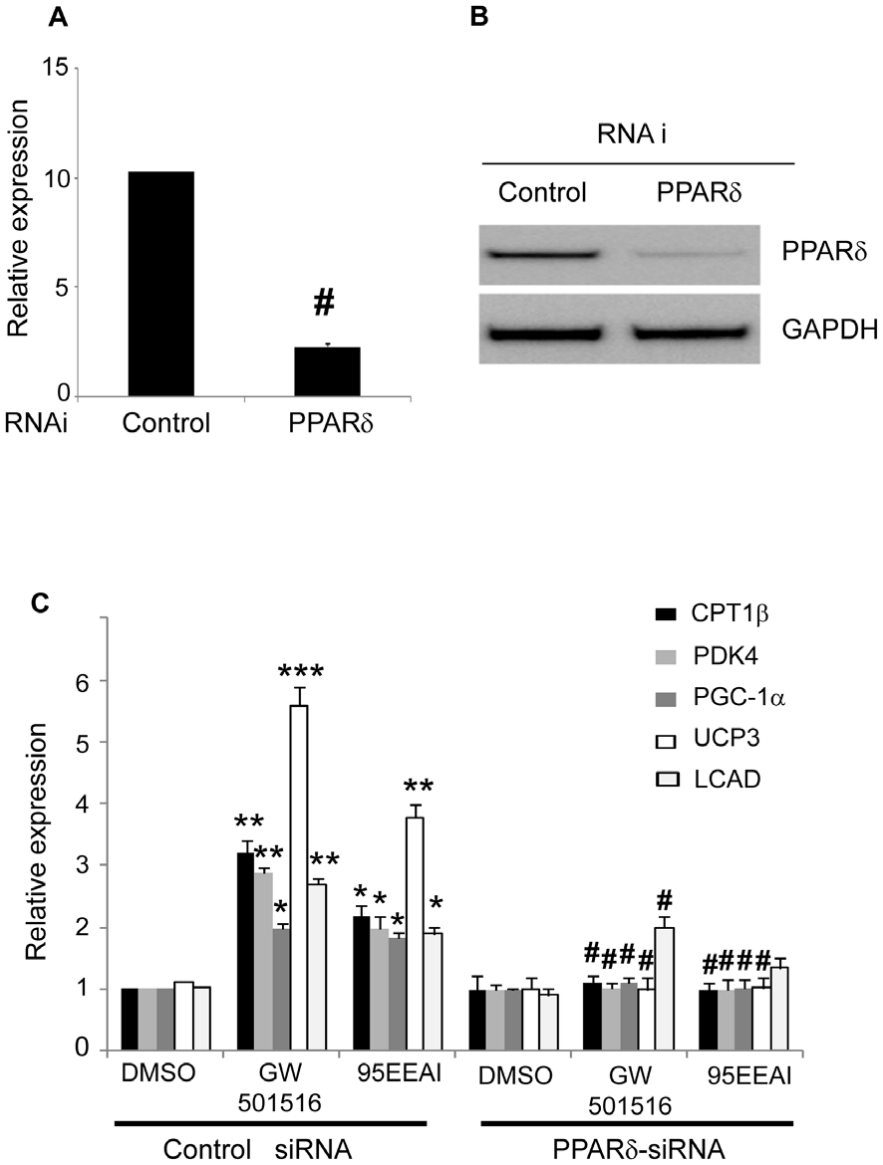
95EEAI-induced effects on PPARδ target gene expression in primary myotubes. Primary myotubes were transfected with PPARδ siRNA pool or control siRNA. At 48 h post-transfection, the cells were treated with 100 nM GW501516, 25 µg/ml 95EEAI or DMSO for 24 h. Cells were harvested for real-time quantitative PCR (A, C) or western blotting (B) GAPDH RNA was used as an internal control for calculating mRNA fold changes. Values are expressed mean ± S.D. from three independent experiments. **, p<0.01; *, p<0.05 for DMSO controls; #, p<0.05 for control siRNA versus PPARδ siRNA. One representative result is shown form three independent western blotting experiments.

We next examined whether a 95EEAI could induce PPARδ-regulated genes in human skeletal muscle cells (Figure S1). To ensure that effects are PPARδ-dependent, we also determined the effect of 95EEAI on the expression of PPARδ target genes in muscle cells in which endogenous PPARδ expression was knocked down with RNAi. As shown in Figure 3A, mRNA expression of PPARδ was reduced by treatment with RNAi. A 95EEAI treatment resulted in induction of several PPARδ target genes involved in the fatty acid oxidation pathway, uncoupling and mitochondrial biogenesis including CPT1b, which cataliyzes the esterification of acyl-CoA to form acyl-carnitine, the rate-limiting step of fatty acid oxidation; PDK4, which plays an important role in switching the fuel source from glucose to fatty acids by inactivating pyruvate dehydrogenase; PGC1α, which is the key mitochondrial transcription regulators; UCP3 which is involved in energy expenditure; and LCAD, which is involved in mitochondrial fatty acid oxidation (Figure 3B). The upregulation of these genes was completely abolished when PPARδ expression was knocked down (Figure 3B). Either WEAI or 50EEAI had no detectable effects on induction of these PPARδ target genes in the same assay (data not shown). These results demonstrated that activation of PPARδ by a 95EEAI leads to elevated expression of genes involved in lipid catabolism.

### A 95EEAI promotes fatty acid oxidation and glucose uptake

**Figure 4:**
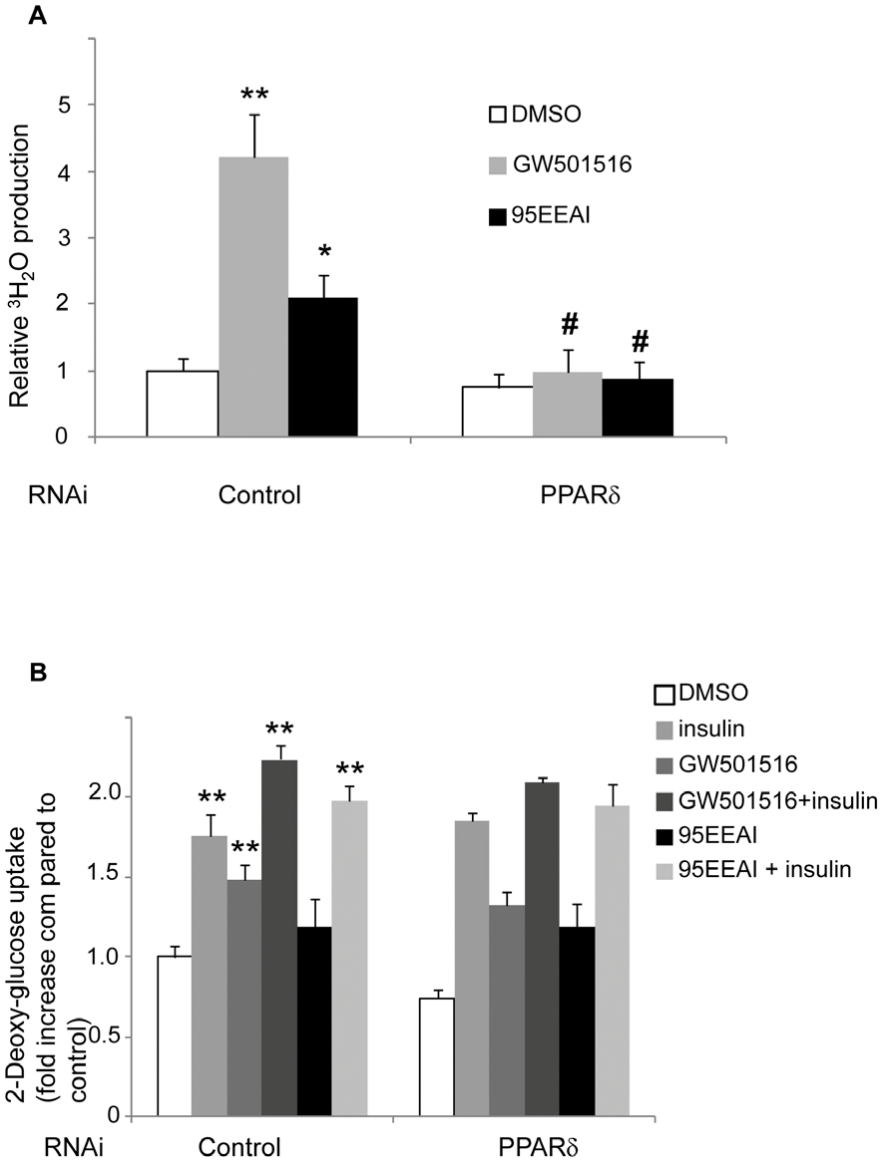
Effects of 95EEAI on fatty acid oxidation and glucose uptake in primary myotubes. Primary myotubes were transfected with PPARδ siRNA or control siRNA. At 48 h post-transfection, the cells were with 100 nM GW501516, 25 µg/ml 95EEAI or DMSO for 24 h. (A) Cells were changed to serum-free medium containing [^3^H]palmitic acid. ^3^H_2_O production was assayed 4 h after incubation. (B) Cells were treated with or without 100 nM insulin at 37°C for 10 min. Glucose uptake assays were conducted as described in Materials and Methods. The cpm results were normalized with protein concentrations. Fold increases in insulin-stimulated glucose uptake were normalized to DMSO-treated control cells. Data shown are means ± S.D. and were obtained from six independent experiments carried out in triplicate. **, p<0.01; *, p<0.05 compared to DMSO control; #, p<0.05 for control siRNA versus PPARδ siRNA.

To determine whether 95EEAI has direct effects on fatty acid oxidation and glucose uptake, we treated differentiated human skeletal muscle cells with 95EEAI. Treatment of 95EEAI led to 2-fold increase in fatty acid oxidation (p<0.05, Figure 4A). The effects of 95EEAI on fatty acid oxidation were completely prevented by the knockdown of PPARδ (Figure 4A).

PPARδ agonist GW501516 is known to enhance basal and insulin-stimulated glucose uptake in human skeletal muscle [14]. As shown in Figure 4B, there was a 1.8-fold increase in insulin-stimulated glucose uptake in 95EEAI-treated cells compared with untreated control cells. However, in the absence of insulin stimulation the increase in glucose uptake in 95EEAI treated cells was not statistically significant. The siRNA-mediated reduction of PPARδ expression was without effect on the stimulation of glucose uptake by either GW501516 or 95EEAI (Figure 4B).

### A 95EEAI attenuates HFD-induced obesity in mice

**Figure 5:**
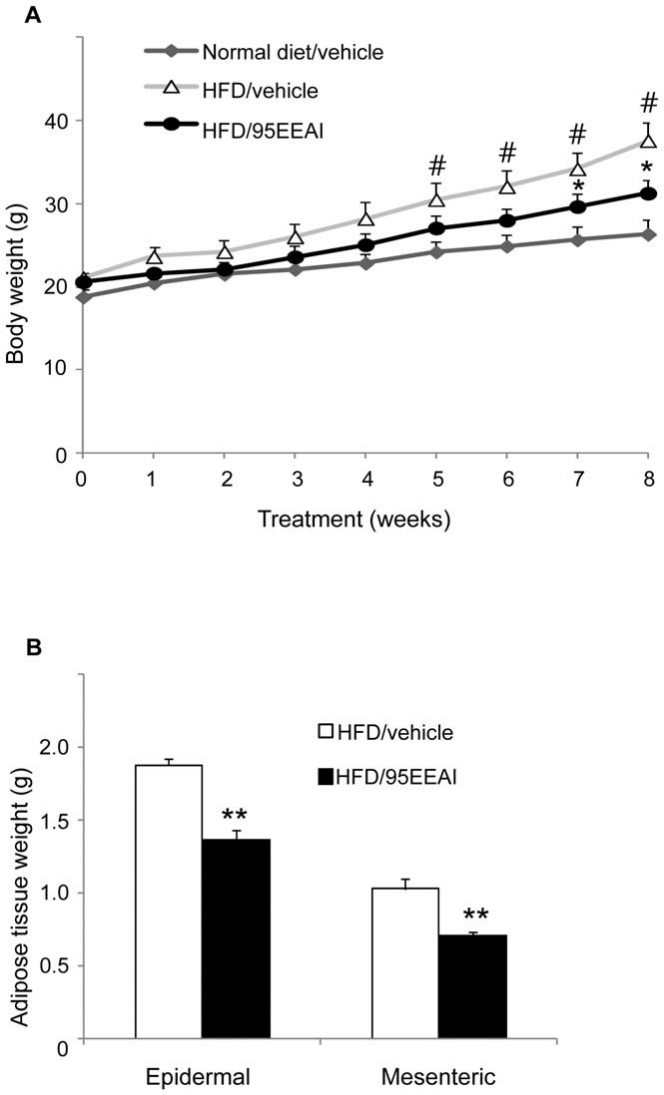
A 95EEAI attenuates body weight gain and adiposity in HFD-fed C57BL/6J mice. Male C57BL/6J mice were fed a normal diet and vehicle, HDF and vehicle or HFD and 200 mg/kg/day 95EEAI for 8 weeks (A) Body weight change. (B) Adipose tissue weight. Values are mean ± S.D. of nine mice. #, p<0.01 compared to normal diet/vehicle group; **, p<0.01; *, p<0.05 compared to HFD/vehicle group.

We and others predicted that enhanced fatty acid utilization and energy expenditure would protect against diet-induced obesity [6]. To determine whether 95EEAI regulate the progression of obesity, mice fed a HFD were orally administered the vehicle or 200 mg/kg 95EEAI once daily for 8 weeks. At the end of 8 weeks high-fat diet feeding, male C57BL/6J mice showed a significant increase in the rate of body weight gain compared with animals fed a normal diet (terminal body weight: normal diet group, 26.3±1.8 g vs. HFD group, 37.6±2.1 g, p<0.01, n = 9; Figure 5A). In contrast, 95EEAI treatment of mice resulted in significantly reduced body weight gain and fat pad mass compared with the vehicle-treated mice on HFD (Figure 5A, B) without reducing food consumption.

**Figure 6:**
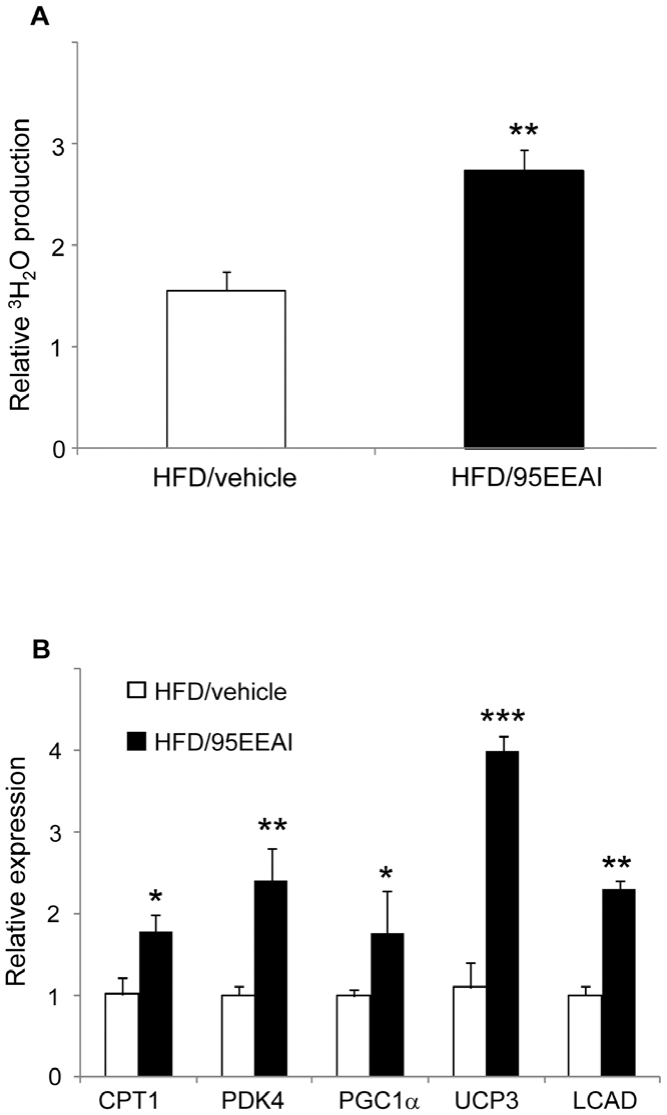
A 95EEAI increases skeletal muscle fatty acid oxidation in C57BL/6J mice. Male C57BL/6J mice were fed a HDF and vehicle or HFD and 200 mg/kg/day 95EEAI for 8 weeks (A) fatty acid oxidation in skeletal muscle (B) Quantitative real-time PCR analysis in skeletal muscle. Values are mean ± S.D. of nine mice. ***, p<0.005; **, p<0.01; *, p<0.05 compared to HFD/vehicle group.

To verify whether 95EEAI stimulates fatty acid oxidation, we measured the levels of fatty acid oxidation in quadriceps muscle. 95EEAI-treated mice on an HFD displayed higher levels of fatty acid oxidation than that of vehicle-treated mice on an HFD (Figure 6A).

We then examined whether increased enhanced fatty acid oxidation in the skeletal muscle affected the levels of lipid-derived substrates, such as nonesterified fatty acids (NEFAs), triglyceride (TG), and ketone bodies in the circulation. The plasma levels of free fatty acids and ketone bodies were significantly lower in 95EEAI-treated mice on an HFD than vehicle-treated mice on an HFD (Table 1). However, there was no statistically significant decrease in the plasma TG levels (Table 1).

**Table 1:** Physical and metabolic parameters in 95EEAI- and vehicle-treated mice.

	Normal diet/vehicle	HFD/vehicle	HFD/95EEAI
Body weight (g)	26.3±1.8	37. 6±2.2#	31.3±0.76*
Liver weight (g)	1.08±0.1	1.09±0.09	0.98±0.04
Epidermal fat weight (g)	0.6±0.03	1.88.1±0.04#	1.37±0.07**
Mesenteric fat weight (g)	0.22±0.02	1.03±0.06#	0.71±0.03**
Food intake (g/day)	3.25±0.8	2. 68±0.9	2. 6±0.5
Glucose (mg/dl): fasted	110. 8±2.9	172.3±15.2	186.±11.1
TG (mmol/l)	0.64±0.7	0.61±0.4	0.58±0.3
NEFA (mEq/l)	0.31±0.01	0.52±0.01#	0. 37±0.02*
Ketone body (µmole/l)	200.3±56.4	221.3±30.1	121.2±12.1**
3-hydroxy-butylate (µmole/l)	197.3±34.2	191. 9±28.7	130.2±12.1**
Total cholesterol (mmol/l)	2.01±0.11	2.51±0.09	2. 63±0. 1

A 95EEAI (200 mg/kg) was orally administrated to mice once daily for 8 weeks.

Values are mean ± S.D. of nine mice.

# , p<0.01 compared to normal diet/vehicle group;

** , p<0.01;

* , p<0.05 compared to HFD/vehicle group.

To confirm that 95EEAI-induced fatty acid oxidation in skeletal muscle *in vivo* was also associated with increased expression of PPARδ target genes involved in fatty acid oxidation pathway, we analyzed the expression of some representative genes (CPT1, LCAD, UCP2, UCP3, and PGC1α) by real-time quantitative PCR assay. The levels of mRNA expression were determined in individual animals, and the average expression for each group was presented. Consistent with results from the primary human myotubes, 95EEAI induced mRNA levels of these genes in skeletal muscle (Figure 6B).

## Discussion

*Artemisia* species are widely used in traditional medicine in East Asia, and have been reported to show anti-obesity, anti-diabetic, anti-lipogenic and anti-hyperglycaemic effects [15]–[20]. However, the molecular mechanism involved in *Artemisia*-induced lipid/carbohydrate metabolism is poorly understood. Since PPARδ activators have been shown to improve insulin resistance and reduce plasma glucose in rodent models of type 2 diabetes and reduce serum triglycerides in sedentary human [21], [22], we aimed to test whether AI extracts have the ability to activate PPARδ as a potential mechanism of action in mediating its beneficial effects.

We showed that 95EEAI interacted with the PPARδ LBD leading to its activation. A 95 EEAI increased the expression of genes involved in lipid catabolism, enhanced fatty acid oxidation and insulin-stimulated glucose uptake *in vivo* as well as in human skeletal muscle cells, protected against diet-induced obesity. Furthermore, in PPARδ knockdown cells, the positive effects of 95EEAI on fatty acid oxidation and their related genes expression were no longer observed, suggesting that 95EEAI-mediated lipid metabolism would be PPARδ-dependent. However, knockdown of PPARδ expression did not alter the 95EEAI-mediated increase in insulin-stimulated glucose uptake. This result is in line with the report by Kramer *et al*. suggesting that direct activation of PPARδ itself is not necessary for the stimulation of glucose uptake [23]. *In vivo* study, administration of 95EEAI to mice fed a HFD had no effect on fasting levels of blood glucose (Table 1). Our finding is in line with the work of Tanka *et al.* [24] who was also unable to detect changes in blood glucose levels in PPARδ agonist-treated mice, despite the marked improvement in glucose tolerance and insulin sensitivity. Brunmair *et al.* [25] have reported that activation of PPARδ acts to suppress glucose utilization as a result of a switch in substrate preference from carbohydrates to lipids in skeletal muscle, thus PPARδ agonist fails to exert any effect on glucose uptake. Lee *et al.* [26] suggest that the improved glucose tolerance and insulin sensitivity triggered by PPARδ agonist is due to promoting an increase in glucose flux through the pentose-phosphate pathway and enhancing hepatic fatty acid synthesis. More studies are needed to elucidate the exact relationship between glucose utilization and 95EEAI-induced PPARδ activation

During starvation, glucose uptake and oxidation are reduced rapidly in muscle, which shifts to use free fatty acids and ketone bodies. In this study, 95EEAI-treated mice on an HFD showed a significant decrease in the plasma levels of free fatty acids and ketone bodies (Table 1). Tanka *et al.* [24] showed that the changes in gene expression by PPARδ agonist are very similar to the gene expression profile induced by fasting in skeletal muscle. Hence, we speculate that the changes in levels of ketone bodies may be attributed to, at least in part, an increased uptake of ketone bodies in muscle through an activation of PPARδ by 95EEAI.

The major compounds isolated from *Artemisia* species include terpenoids, flavonoids, coumarins, acetylenes, caffeoylquinic acids, and sterols [27] (Table S1). Major compounds of 95EEAI had no detectable effect on activation of PPARδ protein (data not shown). Saturated and unsaturated fatty acids, such as arachidonic acid and eicosapentaenoic acid, are reported to be natural ligands for PPARδ [28], [29]. These fatty acids bind and activate PPARδ in the low micromolar range. Although the bioactive component from 95EEAI for activation of PPARδ was not chemically characterized yet, its structure may be similar to that of fatty acids.

Since activation of PPARδ has shown to exert beneficial effects on preventing obesity-related diseases [30], natural compounds that enhance the activity of PPARδ will provide a potential to develop a functional food with anti-obesity and anti-diabetic efficacies.

In summary, our data provide experimental evidence that 95EEAI is a natural PPARδ agonist that robustly induces genes involved in fatty acid metabolism and activates fatty acid oxidation *in vitro* and *in vivo*, suggesting its potential as interventive and preventive measures for the treatment of metabolic disorders.

## Supporting Information

Figure S1 **The effects of 95EEAI on PPARδ target genes expression in primary human myotubes.** Primary human myotubes were treated with different doses of 95EEAI (0, 10, 25, 100 µM) or DMSO for 24 h. Total RNA was extracted from cells, and the mRNA levels of CPT1 and PDK4 genes were quantified by a real-time RT-PCR. Data from 3 independent experiments are represented as means ± S.D. of the relative calculated with GAPDH as standard. *, p<0.05; **, p<0.01 *versus* DMSO-treated cells.
(TIF)

Table S1 **Major chemical compounds in 95% ethanol extracts of** ***Artemisia iwayomogi*** **tested identified by GC-MS.** The 95% ethanol extracts of *Artemisia iwayomogi* were analyzed using the Thermo Scientific TRACE GC Ultra™ gas chromatograph. It was fitted with a split-splitless injector and connected to an MS PolarisQ-Quadrupole Ion Trap (Thermo Electron) fused silica column VB5 (5% phenyl, 95% methylpolyxiloxane, 30 m with 0.25 mm i.d. film thickness 0.25 µm) (J & W Scientific Fisons, Folsom, CA, USA). The injector and interface were operated at 250 and 300°C, respectively. The oven temperature was programmed as follows: 50°C raised to 250°C (4°C/min) and held for 3 min. Helium was the carrier gas at 1 ml/min. The sample (1 µl) was injected in the split mode (1∶20). MS conditions were as follows: ionization voltage EI of 70 eV, mass range 10–350 amu. The components were identified by comparing their relative retention times and mass spectra with those of authentic samples (analytical standards from data base).
(DOC)
