# Supplementary material for: An Ethanol Extract of Artemisia iwayomogi Activates PPARδ Leading to Activation of Fatty Acid Oxidation in Skeletal Muscle
Source: PLoS One. 2012 Mar 27;7(3):e33815. doi: 10.1371/journal.pone.0033815 (PMC3313949; doi:10.1371/journal.pone.0033815)
Supplement: Table S1 — Major chemical compounds in 95% ethanol extracts of Artemisia iwayomogi tested identified by GC-MS. The 95% ethanol extracts of Artemisia iwayomogi were analyzed using the Thermo Scientific TRACE GC Ultra™ gas chromatograph. It was fitted with a split-splitless injector and connected to an MS PolarisQ-Quadrupole Ion Trap (Thermo Electron) fused silica column VB5 (5% phenyl, 95% methylpolyxiloxane, 30 m with 0.25 mm i.d. film thickness 0.25 µm) (J & W Scientific Fisons, Folsom, CA, USA). The injector and interface were operated at 250 and 300°C, respectively. The oven temperature was programmed as follows: 50°C raised to 250°C (4°C/min) and held for 3 min. Helium was the carrier gas at 1 ml/min. The sample (1 µl) was injected in the split mode (1∶20). MS conditions were as follows: ionization voltage EI of 70 eV, mass range 10–350 amu. The components were identified by comparing their relative retention times and mass spectra with those of authentic samples (analytical standards from data base). (DOC) [file pone.0033815.s002.doc]

**Supporting Information**

Table S1. Major chemical compounds in 95% ethanol extracts of *Artemisia iwayomogi* tested identified by GC-MS.

| **Major compounds** | **Retention time** | **%** |
| --- | --- | --- |
| Dibromo-4-methoxybiphenyl | 6.61 | 3.55 |
| Camphor | 16.98 | 41.87 |
| Glycocholic acid | 36.99 | 1.80 |
| Bornane | 51.55 | 5.33 |
| Enopyranoside, derive. | 54.28 | 36.80 |
